# Integrate CRISPR/Cas9 for protein expression of HLA-B*38:68Q via precise gene editing

**DOI:** 10.1038/s41598-019-44336-7

**Published:** 2019-05-30

**Authors:** Yuxin Yin, Elaine F. Reed, Qiuheng Zhang

**Affiliations:** UCLA Immunogenetics Center, Department of Pathology & Laboratory Medicine, Los Angeles, 90095 USA

**Keywords:** Allotransplantation, Genetic testing

## Abstract

The determination of null- or low-expressed HLA alleles is clinically relevant in both hematopoietic stem cell transplantation and solid organ transplantation. We studied the expression level of a questionable (Q) HLA-B*38:68Q allele, which carries a 9-nucleotide (nt) deletion at codon 230–232 in exon 4 of HLA-B*38:01:01:01 using CRISPR/Cas9 gene editing technology. CRISPR/Cas9 gene editing of HLA-B*38:01:01:01 homozygous EBV B cell line resulted in one HLA-B*38:68Q/B*38:01:01:01 heterozygous and one HLA-B*38:68Q homozygous clone. Flow cytometric analysis of monoclonal anti-Bw4 antibody showed the protein expression of HLA-B*38:01:01:01 in homozygous cells was 2.2 fold higher than HLA-B*38:68Q/B*38:01:01:01 heterozygous cells, and the expression of HLA-B*38:68Q/B*38:01:01:01 heterozygous cells was over 2.0 fold higher than HLA-B*38:68Q homozygous cells. The HLA-B*38:68Q expression was further confirmed using anti-B38 polyclonal antibody. Similarly, the expression of the HLA-B*38:01:01:01 homozygous cells was 1.5 fold higher than that of HLA-B*38:68Q/B*38:01:01:01 heterozygous cells, and the HLA-B*38:68Q/B*38:01:01:01 heterozygous cells was over 1.6 fold higher than that of HLA-B*38:68Q homozygous cells. The treatment of HLA-B*38:68Q homozygous cells with IFN-γ significantly increased its expression. In conclusion, we demonstrate that HLA-B*38:68Q is a low-expressing HLA allele. The CRISPR/Cas9 technology is a useful tool to induce precise gene editing in HLA genes to enable the characterization of HLA gene variants on expression and function.

## Introduction

Immune recognition of infectious pathogens is mediated through the presentation of foreign peptides by the Human Leukocyte Antigen (HLA) molecules^1^. The HLA class I (HLA-A, -B and -C) and class II (HLA-DR, -DQ and -DP) are the most polymorphic genes in the human genome with 18,771 different HLA alleles identified (IMGT/HLA v3.33.0)^2^. The high level of HLA polymorphism reflects its biological importance to present a large diversity of peptides to the immune system and trigger effective immune responses^3^. In allogeneic hematopoietic stem cell transplantation (HSCT), HLA mismatches between donor and recipient leads to strong alloimmune responses that result in either graft versus host disease (GVHD) or engraftment failure^4–6^.

Mutations such as insertions, deletions in HLA sequences can lead to the introduction of stop codons and/or incorrectly spliced products that result in aberrant expressed alleles. HLA null alleles (N) are characterized by the lack of antigen expression of the HLA molecule on cell surface^2^. Failure to identify HLA null alleles in donors may result in HLA mismatches that can stimulate allogeneic T cells and trigger GVHD in HSCT^7^. A ‘Q’ suffix is used for HLA alleles that their expressions have not been confirmed and remain ‘Questionable’. In HSCT, if the presence of a questionable allele in the donor is in fact a null allele, mistyping the null allele results in an antigen mismatch between the donor and the recipient and increases the risk of GVHD^7^. On the contrary, mistyping of a lowly expressed HLA allele as a null allele in the recipient can lead to inaccurate donor selection and result in GVHD as well. Therefore, questionable alleles require confirmation of their actual HLA expression level. For example, HLA-A*23:19Q has a single polymorphism at position 619 (G > A) compared to HLA-A*23:01:01. Gerritsen *et al*. demonstrated that HLA-A*23:19Q has no expression by serology and flow cytometry, therefore it should be reclassified as a null allele^8^. Hirv *et al*.^9^ reported a patient, who carried HLA-A*30:14L and HLA-A*02:01 alleles, was mistyped as HLA-A*02:01 homozygous. The mistyping of HLA-A*30:14L led to severe GVHD in the patient transplanted with HSCT from an HLA-A*02:01 homozygous donor. Currently, there are 663 null alleles (N), 44 questionable alleles (Q), 5 low expression alleles (L), and 1 soluble alleles (S) identified in IMGT/HLA data base^2^. The HLA matching of the questionable alleles between the donor and recipient remains challenging. Therefore, it is important to determine the expression patterns of abnormally expressed HLA variants^7^.

The CRISPR (clustered regularly interspaced short palindromic repeats) is an adoptive immune system in bacteria that protects the bacterium from invading foreign genetic elements such as plasmid and bacteriophages^10^. The CRISPR/Cas9 system contains two components: a guide RNA (gRNA) and a CRISPR-associated endonuclease (Cas protein)^11,12^. The gRNA is a short RNA composed of a scaffold sequence needed for Cas-binding and a user-defined ∼20 nucleotide spacer that defines the genomic target to be modified^13^. The gRNA spacer sequence could be designed to target DNA sites with Protospacer Adjacent Motif (PAM)^14,15^. The most common PAM sequence recognized by Cas9 is NGG that is found directly downstream of the target DNA. The CRISPR/Cas9 cuts double strand DNA to generate double strand breaks (DSBs) between 3–4 bp upstream of the NGG PAM under the guidance of gRNA^16^. The DSBs can be repaired by non-homologous end joining (NHEJ), which is an error-prone process that introduces unpredictable insertions and deletions (indels); DSBs can also be repaired by homology directed repair (HDR) with the presence of DNA template, which induces desired DNA editing^11,12,17^. Two types of the DNA template can be used for HDR: a small single stranded DNA (ssDNA) oligonucleotide with 30–67 nt homology arms flanking the gene editing site^18^ or a double stranded DNA (dsDNA) plasmid with long homology arms of 1–3 kb^19^.

The recent discovery of CRIPSR/Cas9 system provides a faster and more economical approach to gene editing^20^ compared to the traditional zinc-finger nucleases (ZFNs)^21^ and transcription activator-like effector nuclease (TALEN) methods^22^. The goal of this study was to generate homozygous and heterozygous cells carrying the HLA-B*38:68Q with deletion at codon 230–232 at exon 4 using CRISPR/Cas9 gene editing to study the effect of this mutation on HLA-B*38:01:01:01 expression.

## Results

### crRNA design and selection

**Figure 1:**
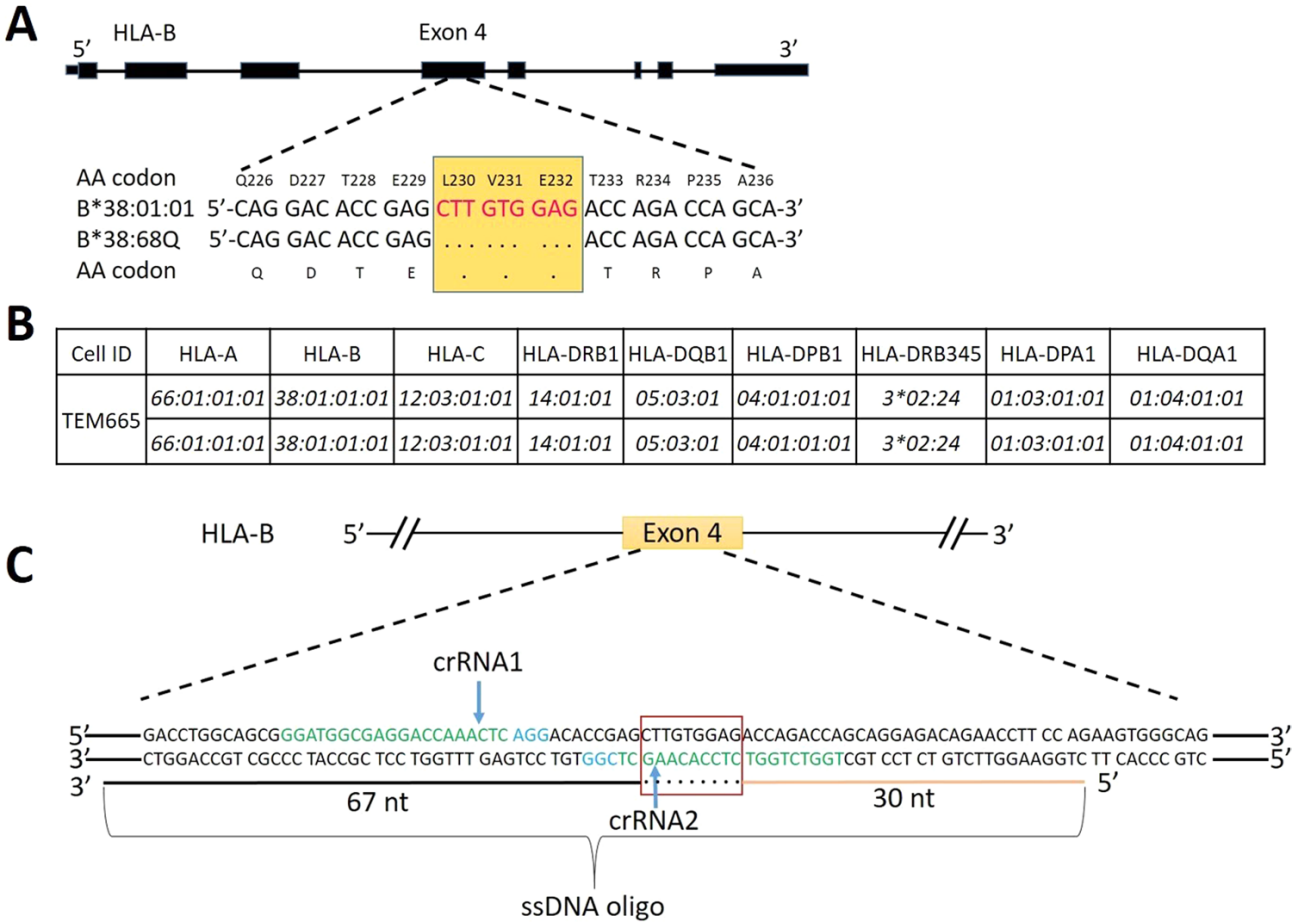
crRNA and ssDNA target design. (**A**) Comparison of HLA-B*38:01:01:01 and HLA-B*38:68Q in nucleotide and protein sequences. Yellow box highlights the 9-nt deletion mutation and “.” indicates the deletion. Numbers above sequence correspond to amino acid codon positions. (**B**) HLA typing of TEM665 homozygous HLA-B*38:01:01:01 EBV transformed B cell line used for gene editing. (**C**) Design of crRNA and ssDNA target on HLA-B*38:01:01:01. crRNA1 and crRNA2 are colored in green. The Protospacer Adjacent Motif (PAM) recognition sites are marked in blue. The red box indicates the location of 9-nt deletion. “.” means that the ssDNA oligo contains the 9-nt deletion. Blue arrows show the position of double strand breaks (DSBs) that should be introduced by CRISPR/Cas9.

We identified a new HLA B allele that is similar to HLA-B*38:01:01:01 except for a nine-nucleotide deletion (5′-CTTGTGGAG-3′) at codon 230 to 232 that results in a coding shift at α3 domain of *HLA-B38* (Fig. 1A). The sequence was submitted to the GenBank database (accession number MF069211) and IMGT/HLA databases (submission number HWS10028807). Since the expression level of this novel B*38 allele is unknown, it was named HLA-B*38:68Q. To determine if the deletion at codon 230–232 affected the level of protein expression, the homozygous HLA-B*38:01:01:01 EBV B cell line TEM665 was used to generate homozygous HLA-B*38:68Q alleles to study its expression (Fig. 1B). GeneArt^TM^ CRISPR Search and Design Tool was used to design crRNAs targeting the DNA sequences close to the 9-nt deletion site at exon 4 of HLA-B*38:01:01:01 (Fig. 1C). crRNA1 (5′-GGATGGCGAGGACCAAACTC-3′) was designed to recognize −12 to −31 bp upstream of the 9-nt deletion, and crRNA2 (5′-TGGTCTGGTCTCCACAAGCT-3′) was designed to recognize −2 to +9 bp sequence of the 9-nt deletion. Both crRNAs share a ssDNA target with a 67-nt of left arm and a 30-nt of right arm of the deletion site (Fig. 1C). The crRNA1 and crRNA2 were then mixed with universal tracrRNA to form gRNA1 and gRNA2. Next, Cas9 protein (1.5 µg) and gRNA1/gRNA2 (360 ng) were mixed to form Cas9 ribonucleoprotein (RNP), respectively^18^. Twenty four electroporation conditions were tested to optimize transportation efficiency using Neon transfection system^23^ (Supplemental Table 1). The program of the highest transportation efficiency (58.4%) was selected for transfecting HLA-B*38:01:01:01 homozygous EBV B cell line. Our results showed that gRNA1 induced 22.6% DSB cleavage and gRNA2 induced 13.8% DSB cleavage using GeneArt® Genomic Cleavage Detection assay. The gRNA1 was chosen for transfection with ssDNA target due to its high efficiency.

### Generation of HLA-B*38:68Q clones using CRISPR/Cas9 gene editing

**Figure 2:**
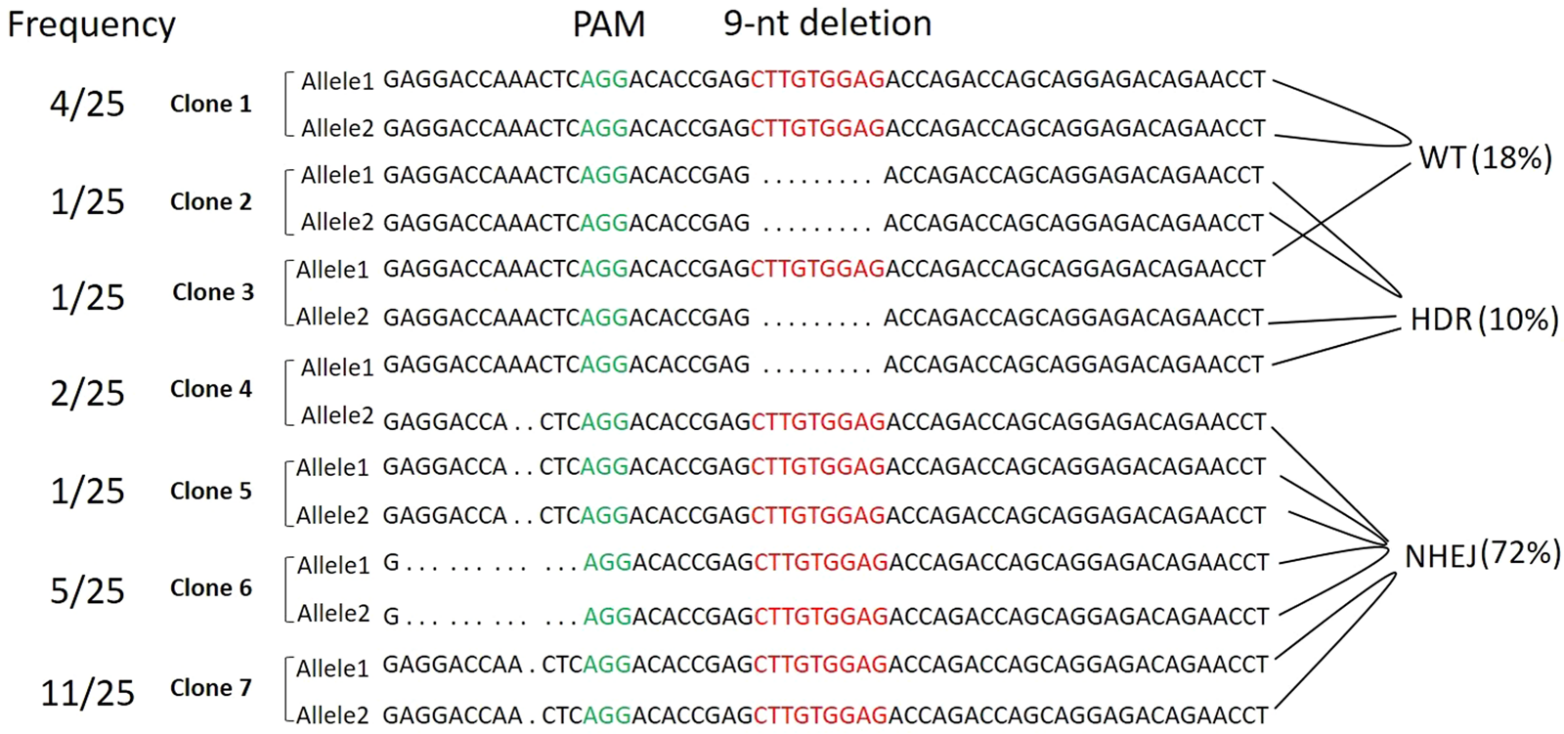
Single cell clones derived by CRISPR/Cas9-mediated gene editing. Nucleotides highlighted in red are the position with the 9-nt deletion. The Protospacer Adjacent Motif (PAM) sequence is highlighted in green. “.” indicates the deletion. The frequency of each single cell derived clone as well as the relative percentage of wild type (WT), NHEJ, and HDR were listed (n = 25).

**Figure 3:**
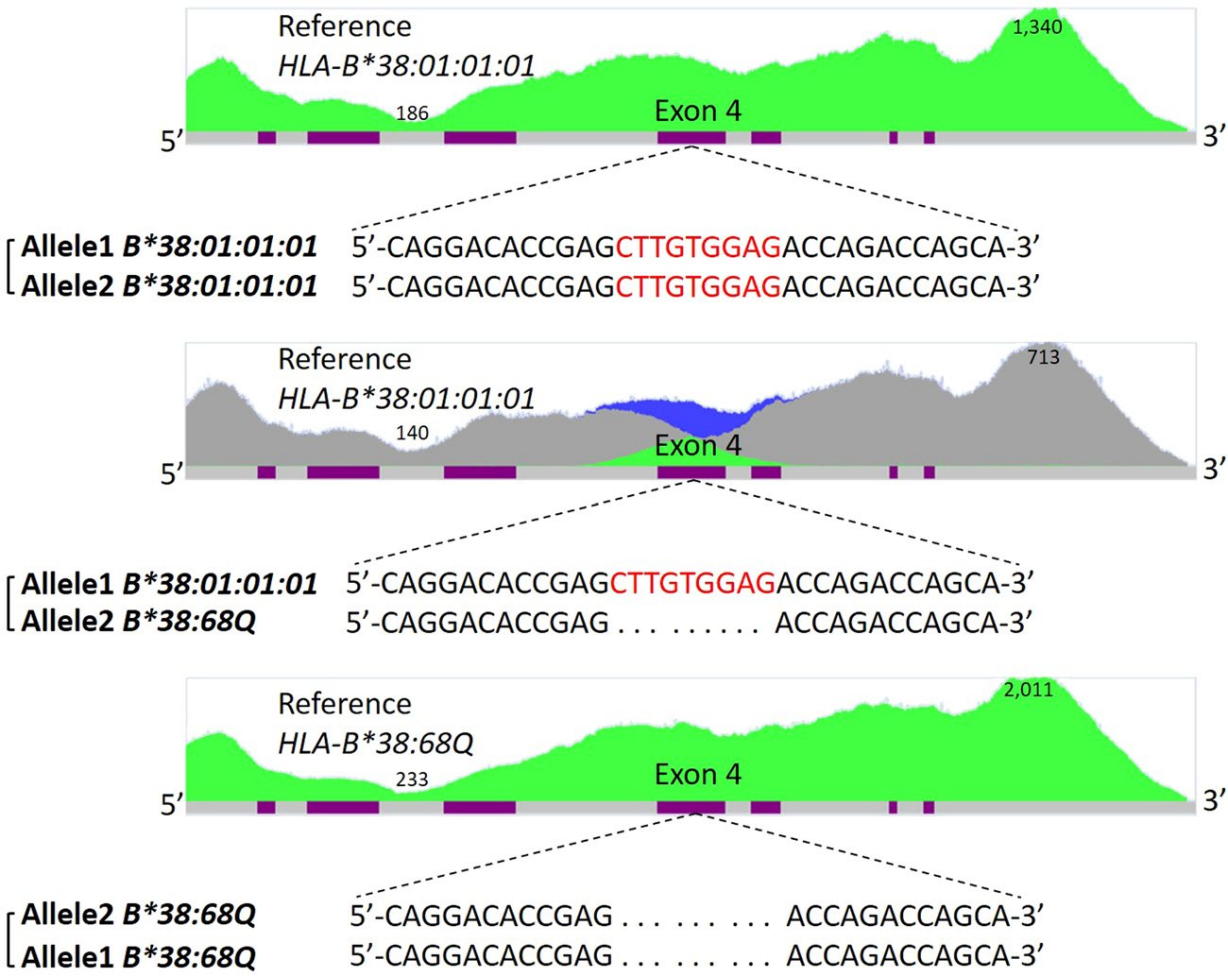
Confirmation of HLA-B*38:68Q gene editing by NGS sequencing. (**A**) HLA-B*38:01:01:01 homozygous; (**B**) HLA-B*38:68Q/B*38:01:01:01 heterozygous; (**C**) HLA-B*38:68Q homozygous. The histograms display NGS read coverage from 5′ UTR to 3′ UTR. The purple color represents exons. The height of the blue (specific reads from one allele), green (homozygous sequences), and grey (sequences shared by both alleles) colors indicates relative read depth. The highest and lowest coverages are shown with numbers. Sequence in red indicates the location of 9-nt, and “.” indicates the 9-nt deletion.

Gene editing of HLA-B*38:01:01:01 homozygous EBV B cell line by CRISPR/Cas9 using gRNA1 resulted in 25 single cell derived clones. The 25 clones were then sequenced by Next Generation Sequencing (NGS) to ensure correct gene editing was achieved (Fig. 2). The CRISPR/Cas9 editing is usually bi-allelic. Our results showed a total of 41 alleles were edited while 9 alleles had no editing. These 41 edited alleles resulted in 21 (84%) modified clones with 7 genotypes (Fig. 2). Of the 25 clones, 5 alleles (10%) were edited through the HDR pathway and 36 alleles (72%) were edited through the NHEJ pathway. The most frequent editing was an A deletion at codon 225 through NHEJ pathway which resulted in 11 (44%) clones with homozygous A deletion (Clone 7, Fig. 2). The CRISPR/Cas9 editing generated 5 HLA-B*38:68Q alleles that resulted in one HLA-B*38:68Q homozygous clone and one HLA-B*38:01:01:01/B*38:68Q heterozygous clone. The sequencing of the entire HLA-B gene from the 5′ UTR to 3′ UTR of the HLA-B*38:68Q homozygous clone and a HLA-B*38:01:01:01/B*38:68Q heterozygous clone showed that other than the correct 9-nt deletion on codon 230–232 at exon 4, there was no non-specific gene editing detected in the rest of the HLA-B*38:68Q alleles in both clones (Fig. 3).

### HLA-B*38:68Q shows inferior cell surface expression by flow cytometry

**Figure 4:**
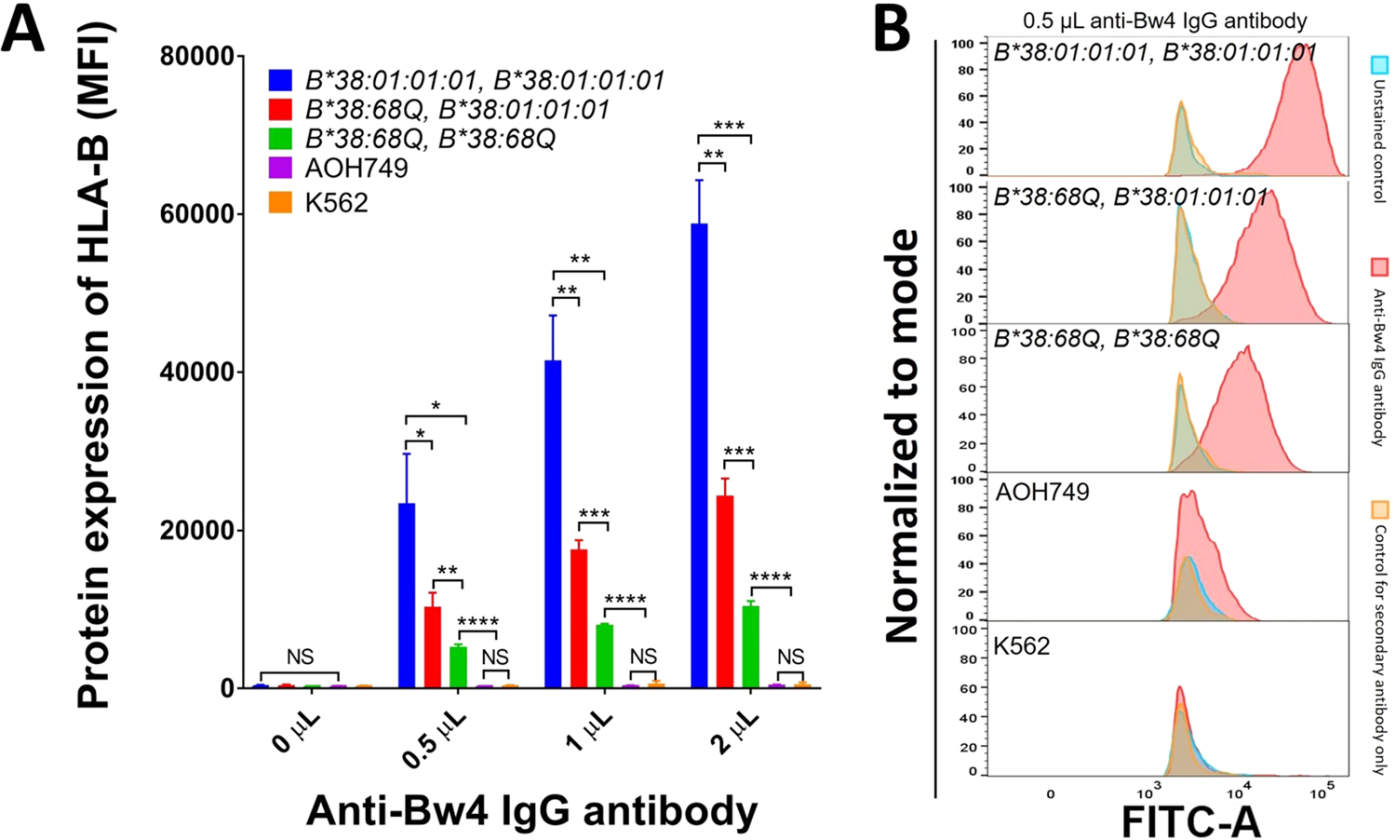
Flow cytometric analysis of *HLA-B38* protein expression using monoclonal antibody. (**A**) HLA-B expression detected in HLA-B*38:01:01:01 homozygous (blue), HLA-B*38:68Q/B*38:01:01:01 heterozygous (red), HLA-B*38:68Q homozygous (green), and negative controls (AOH749 purple and K562 in orange) using anti-Bw4 IgG antibody. Samples were tested in triplicate and the expression level is shown as mean fluorescence intensity (MFI) ± SD. (**B**) Representative histograms of HLA-B expression in HLA-B*38:01:01:01 homozygous, HLA-B*38:68Q/B*38:01:01:01 heterozygous, HLA-B*38:68Q homozygous and negative controls using 0.5 μL of anti-Bw4 IgG antibody. Normalized to mode scales all channels as a percentage of the maximum count. *P < 0.05, **P < 0.01, ***P < 0.001, ****P < 0.0001, and NS indicates the variables are not significant.

Both HLA-B*38:68Q and HLA-B*38:01:01:01 express Bw4 epitope in α1-helix at position 77–83^24^, therefore anti-Bw4 antibody was used to detect the expression levels of HLA-B*38:68Q homozygous and HLA-B*38:68Q/B*38:01:01:01 heterozygous clones. The monoclonal anti-Bw4 antibody does not cross reactive to A66, C12 HLA antigens expressed on the HLA-B*38:01:01:01 homozygous TEM665 EBV B cell line using Luminex Single Antigen Bead (SAB) testing (Supplemental Fig. 1). The K562 cell line lacking HLA molecule expression and the Bw6 homozygous AOH749 EBV B cell line were used as negative controls. The wild type HLA-B*38:01:01:01 homozygous TEM665 EBV B cell line was used as a positive control (Fig. 1B). Flow cytometry analysis showed the median fluorescence intensity (MFI) of antibody binding to HLA-B*38:01:01:01 was at 58,080 ± 5,368 MFI in the HLA-B*38:01:01:01 homozygous TEM665 EBV B cell line compared to 10,383 ± 639 MFI in homozygous HLA-B*38:68Q cells using 2 μL of anti-Bw4 antibody (Fig. 4). As expected, HLA expression was negative in AOH749 and K562 cell line (Fig. 4). These results indicate that HLA-B*38:68Q is expressed on the cell surface, yet at a significantly lower level than the wild type HLA-B*38:01:01:01 molecule. Notably, the expression of HLA-B*38:01:01:01 homozygous cell line was 2.2 fold higher than HLA-B*38:68Q/B*38:01:01:01 heterozygous clones (24,067 ± 2,066 MFI, *P* < *0*.*01*). In addition, the expression of HLA-B*38:68Q/B*38:01:01:01 heterozygous cells was 2.0 fold higher than HLA-B*38:68Q homozygous clone (*P* < *0*.*001*), suggesting HLA-B*38:68Q exhibits a reduction of antigen expression in a gene dose dependent manner (Fig. 4). Similar results were found using 0.5 μL and 1 μL of anti-Bw4 antibody (Fig. 4).

**Figure 5:**
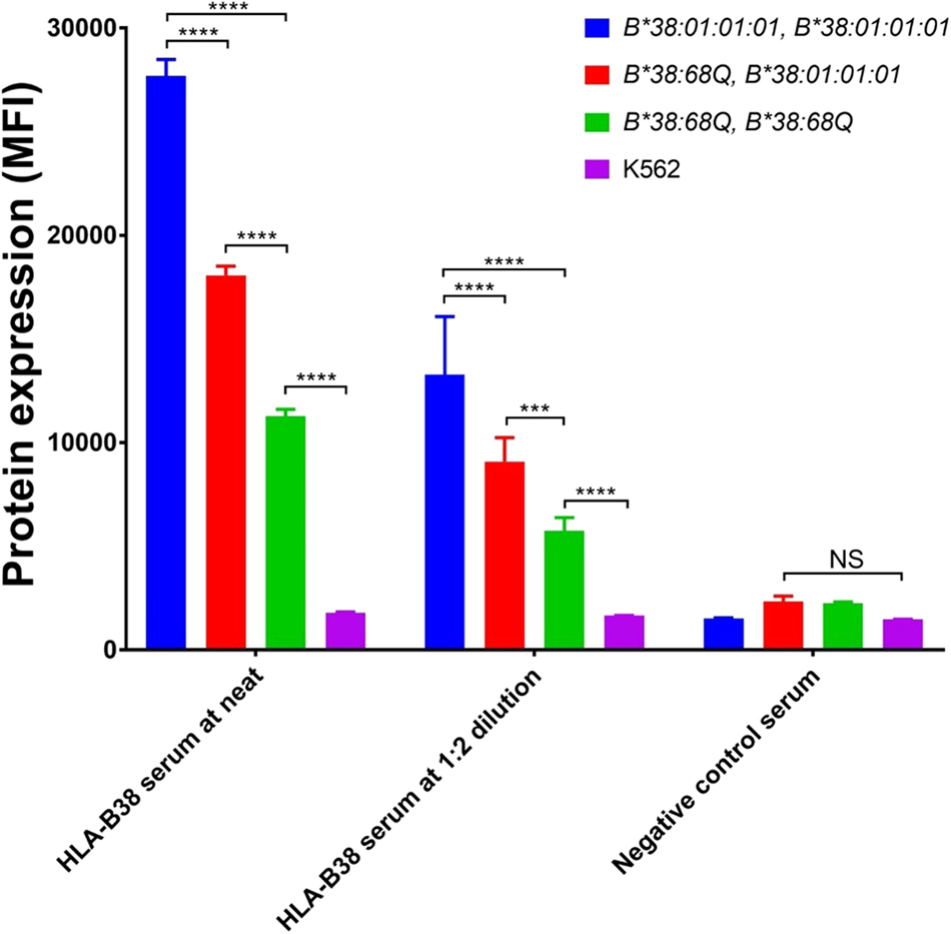
Flow cytometric analysis of *HLA-B38* expression using polyclonal antibody. *HLA-B38* expression is shown in HLA-B*38:01:01:01 homozygous (blue), HLA-B*38:68Q/B*38:01:01:01 heterozygous (red), HLA-B*38:68Q homozygous (green) and K562 negative control (purple) using anti-B38 antibody from UCLA serum exchange. Samples were tested in triplicate and the expression level is shown as mean fluorescence intensity (MFI) ± SD. ***P < 0.001, ****P < 0.0001, and NS indicates the variables are not significant.

The expression of the HLA-B*38:68Q was further confirmed using human polyclonal antibody against *HLA-B38* at neat and at 1:2 dilution. *HLA-B38* antibody recognizes epitopes at 80I and 158 T of the α1 and α2 helixes of HLA class I molecule^25^. The polyclonal anti-B38 antibody does not cross react to A66, C12, DR14, DR52, DQ5 and DP1 HLA antigens expressed on the HLA-B*38:01:01:01 homozygous TEM665 EBV B cell line using Luminex SAB test (Supplemental Fig. 2). Luminex SAB testing showed the MFI of the *HLA-B38* antibody was 22,636 at neat and 14,551 at 1:2 dilution. Our results showed the expression of *HLA-B38* in the HLA-B*38:01:01:01 homozygous cells (27,730 ± 784 MFI) was 1.5 fold higher than that of HLA-B*38:68Q/B*38:01:01:01 heterozygous cells (18,184 ± 387 MFI, *P* < 0.0001), and the HLA-B*38:68Q/B*38:01:01:01 heterozygous cells was over 1.6 fold higher than HLA-B*38:68Q homozygous cells at neat (11,427 ± 190 MFI, *P* < 0.0001, Fig. 5). Similar results were found using anti-B38 antibody at 1:2 dilution. In summary, this data demonstrates that HLA-B*38:68Q is a low-expressed HLA allele, not a null allele.

**Figure 6:**
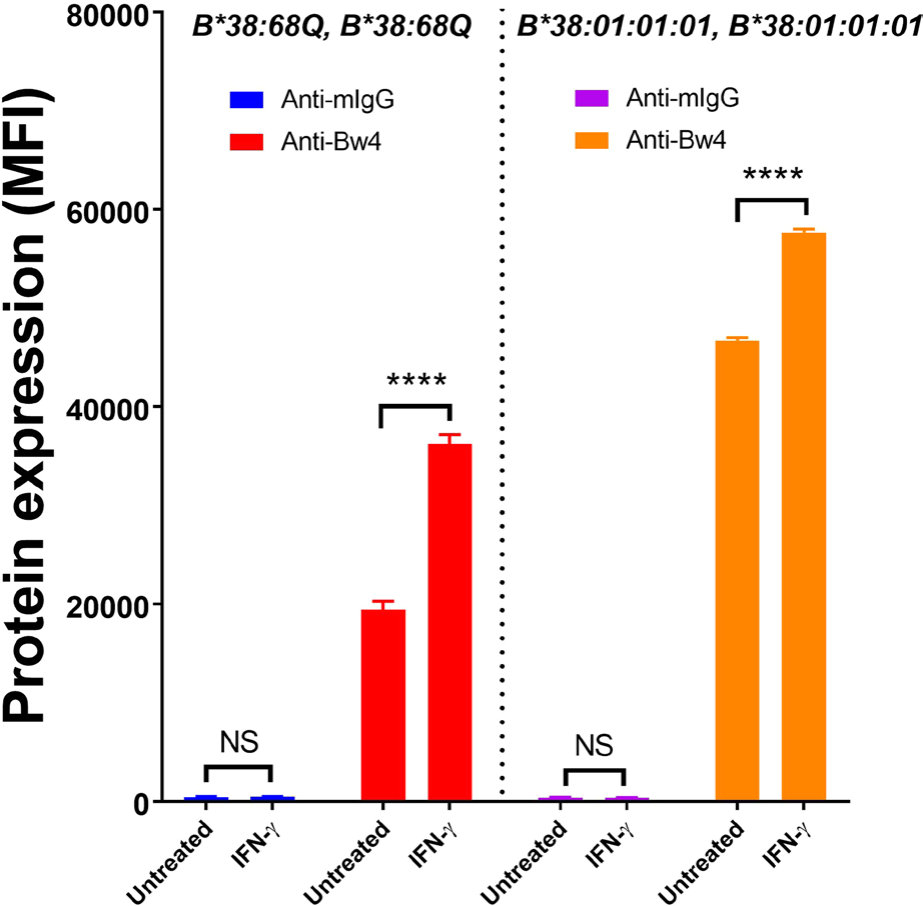
IFN-γ induced *HLA-B38* cell surface expression. HLA-B expression of HLA-B*38:01:01:01 homozygous cell line (untreated in purple and IFN-γ treated in orange) and HLA-B*38:68Q homozygous cell line (untreated in blue and IFN-γ treated in red) was determined using anti-Bw4 IgG antibody. Samples were tested in triplicate and the expression level is shown as mean fluorescence intensity (MFI) ± SD. ****P < 0.0001, and NS indicates the variables are not significant.

Allograft rejection often correlates with increased cytokine production including IFN-γ^26^. We therefore tested if the expression of HLA-B*38:68Q will be stimulated by IFN-γ under inflammatory environment. Our results showed that in the HLA-B*38:68Q homozygous cells, the treatment of IFN-γ significantly increased the HLA-B expression and resulted in a binding of 36,036 ± 887 MFI, 1.9 fold higher than the HLA-B*38:68Q homozygous cells without IFN-γ treatment (19,379 ± 900 MFI, *P* < 0.0001, Fig. 6). Similarly, in the HLA-B*38:01:01:01 homozygous cells, the expression of HLA-B treated with IFN-γ was at 57,646 ± 357 MFI, which was 1.2 fold higher than the untreated group (46,642 ± 231 MFI, *P* < 0.0001). The results showed that although the expression of HLA-B*38:68Q can be upregulated by IFN-γ treatment, the upregulation of HLA-B expression was much lower compared to IFN-γ treated HLA-B*38:01:01:01 homozygous cells (Fig. 6).

## Discussion

In this study, we reported efficient HLA-B*38:01:01:01 gene modification and expression in an EBV B cell line using the CRISPR/Cas9 system. We successfully introduced gene editing in 84% of clones and achieved precise deletion at codon 230–232 at exon 4 in 5 alleles. Similar to other publications, the CRISPR/Cas9 gene editing of HLA-B*38:01:01:01 involved DNA repair via either NHEJ or HDR pathway^27^. However, even in the presence of guided DNA templates, 72% of gene editing was through the NHEJ repair pathway compared to 10% in HDR pathway. HDR pathway provides desired repair of the target DNA in the presence of template DNA. The low incidence of HDR makes the selection of precise gene editing challenging. In order to achieve higher HDR gene editing efficiency, the DSBs induced by CRISPR/Cas9 nuclease should be in close proximity to the edit site^18^. The homologous recombination rate could be increased with larger flanking sequences, therefore standard gene deletion/disruption protocols typically use flanking regions over 1 kb on either side of the target gene to increase HDR^28^. The evidence of using cell lines deficient in NHEJ pathways increased the levels of HDR suggesting these two pathways are competitive^29^. Recent studies have demonstrated the use of KU70, KU80 or DNA ligase IV to suppress key NHEJ molecule can increase HDR pathway^30^.

Our study successfully demonstrated that the deletion at codon 230–232 at exon 4 in HLA-B*38:01:01:01 reduced *HLA-B38* antigen expression on the cell surface using CIRSPR/Cas9 system. The expression of the HLA-B*38:01:01:01 homozygous allele was 2.2 fold higher than HLA-B*38:68Q/B*38:01:01:01 heterozygous cells. Further, HLA-B*38:68Q/B*38:01:01:01 protein expression in heterozygous cells was >2.0 fold higher compared to HLA-B*38:68Q homozygous cells. Similar results were found using the anti-B38 polyclonal antibody. These results suggested that the deletion of three amino acids (LVE) at exon 4 of HLA-B*38:01:01:01 reduces the protein expression level. In addition, our data also showed that after IFN-γ treatment, the expression of HLA-B*38:68Q was significantly increased compared to the untreated cells. These results suggested that HLA-B*38:68Q may still play an important role in allograft rejection^31^. However, the expression increase of the HLA-B*38:68Q with the IFN-γ treatment was much lower compared to the HLA-B*38:01:01:01 homozygous cells. The cause of the reduced expression of HLA-B*38:68Q allele is unclear and requires additional study. However, possible mechanisms that proposed for lower HLA expression including aberrant dominate splice site, such as A*24:02:03Q^32^ and HLA conformation alterations caused by disulfide bond changes, such as A*32:11Q^33^.

Currently, there are 44 questionable HLA alleles (Q) in IMGT/HLA data base^2^. The frequency of these questionable alleles has not been well established, particularly the HLA allele frequency is largely based on the Caucasian population. Therefore, the frequency of these questionable alleles may be underestimated in other populations. With the advancement of the full gene HLA sequencing by NGS technology, the laboratory is able to obtain high resolution HLA typing with minimum ambiguities. However, due to additional sequencing information on exons outside the antigen recognition sites (ARS) and introns, it is likely that more questionable alleles will be discovered. The knowledge of the expression level of these questionable alleles may be important for donor selection in HSCT. Petersdorf *et al*. demonstrated that increasing expression level of the patient’s mismatched HLA-C allele was associated with increased risk of grades III-IV acute GVHD with odds ratio of 1.34 in HSCT^34^. In addition, the understanding of the expression level can also help to identify donors with the least immunogenic mismatches, or select donors to cross permissive immunological barriers for highly sensitized patients in solid organ transplantation. These questionable alleles could also be potential null alleles. Failure to identify HLA null alleles in donors may cause severe GVHD in HSCT^7^. Increasing knowledge of the expression level of HLA variant alleles will help to improve the understanding of HLA allogenicity in both HSCT and solid organ transplantation. The CRISPR/Cas9 system provides an effective tool to study the expression level of these variant HLA alleles. In addition, CRISPR/Cas9 can also introduce insertions and deletions at the UTRs, exons and introns to study the regulations and functions of HLA genes.

CRISPR/Cas9 has been used to facilitate correction of mutated genes in various diseases. Recently, CRISPR/Cas9 has been used to treat single nucleotide polymorphism in the β-globulin gene to treat sickle cell diseases in a mouse model^35^. The chimeric antigen receptor (CAR) modified T cells have been applied to various cancers, especially in B cell hematologic malignancies^36^. With the application CRISPR/Cas9 system, Liu *et al*.^37^ have successfully down regulated the expression of HLA class I and TCRα to generate a universal chimeric antigen receptor (CAR) T cells. The CRISPR/Cas9 technology has also come under the spotlight in transplantation. Entry of human immunodeficiency virus (HIV) into target cells requires both CD4 and CCR5 receptors^38^. A 32-base pair deletion in CCR5 (CCR5-Δ32) is associated with reduced HIV transmission risk and delayed disease progression^39^. In HIV+ patients with hematological malignancies, gene editing using CRISPR/Cas9^40^ has been used to generate homozygous CCR5-Δ32 deletion in CD34+ cells to introduce HIV resistance. Currently, there are several ongoing clinical trials evaluating the safety of transplantation of CRISPR modified CCR5-Δ32 CD34+ cells in HIV+ patients with hematological malignances^41,42^. In conclusion, the CRISPR/Cas9 system is a powerful gene editing tool that can be used to study HLA gene expression and function and improve HLA matching in hematopoietic stem cell and solid organ transplantation. Modification of HLA gene expression by CRISPR/Cas9 also promises to provide new approaches for cellular therapies in transplantation.

## Material and Methods

HLA-B*38:68Q was submitted to the GenBank database (accession number MF069211) and IMGT/HLA database (submission number HWS10028807) with full genomic allele sequence as a questionable allele due to its unknown surface expression by our center in 2017^43^. Research approval for performing CRISPR/CAS9 on the sample was granted by the UCLA Institutional Review Board (IRB#14–000516).

### Cell lines

An HLA-B*38:01:01:01 homozygous EBV transformed B cell line TEM665 (from International Histocompatibility Workshop, IHW9057, Fig. 1B) was selected for gene editing, and in addition, a Bw6 homozygous EBV B cell line AOH749 (from AOH Workshop, AOH9004) and/or K562 (from UCLA Immunogenetics Center) which lacks HLA expression were used as negative controls for monoclonal or polyclonal antibody test. AOH749 expresses HLA A31, B65, Bw6, C8, DR1, DQ5, DP3 and DP0401, which does not cross reactive to the anti-Bw4 antibody. All cell lines were cultured in RPMI-1640 (GE Healthcare Life Sciences, USA) containing 20% FBS (Omega, USA), 1% Amphotericin B (Corning, USA), 1% Penicillin Streptomycin Solution (Corning, USA), 1% Gibco™ Sodium Pyruvate (100 mM) (ThermoFisher, USA), 1% Gibco™ MEM Non-Essential Amino Acids Solution (ThermoFisher, USA), and 0.1% Gibco™ 2-Mercaptoethanol (55 mM) (ThermoFisher, USA). All cultures were maintained in 5% CO_2_, 37 °C.

### crRNA design and Cas9 Ribonucleoproteins (RNPs) complex formation

Oligonucleotides of crRNA sequences were designed with the GeneArt^TM^ CRISPR Search and Design Tool. crRNAs (Synthesized by ThermoFisher, USA) and tracrRNA from TrueGuide™ Synthetic gRNA kit (ThermoFisher, USA) were re-suspended using DNA Suspension Buffer pH 8.0 (Teknova, USA) to make a concentration of 100 pM. In Brief, the reaction of forming gRNA consisted of 10 μL crRNA, 10 μL tracrRNA, 10 μL annealing buffer, and 20 μL nuclease-fee H_2_O, making a final volume of 50 μL. Then the mixture was incubated at 95 °C for 5 mins followed by 10 mins on 78 °C, and then 25 °C for 5 mins. To make Cas9 RNPs, mixing the following items: 11.25 μL gRNA formed in previous step, 7.5 μL Cas9 Nuclease V2 (5 μg/μL) (ThermoFisher, USA), and 131.25 μL buffer R from Neon kit (Invitrogen, USA), making a final volume of 150 μL. The mixture was incubated at room temperature for 10~20 mins.

### ssDNA target design/Homologous recombination assays

To create homologous recombination (HR) assays, two gRNAs target the sequence near the deletion site within the HLA-B gene were designed and synthesized^18^. The Cas9 RNPs were then used to transfect cells via Neon® electroporation (Invitrogen, USA) for 24-well format electroporation transfection testing for B cell line. The genomic cleavage efficiency was then evaluated using the GeneArt® Genomic Cleavage Detection kit (ThermoFisher, USA) at 48 h post transfection. The cleavage efficiencies were calculated based on the relative agarose gel band intensity, which was quantified using a high sensitivity DNA chip on TapeStation 2200 (Agilent, USA). Cleaved efficiency was calculated following the manufacturer’s instruction. A program with voltage set at 1700 V, pulse width set at 20 ms, and one pulse was used for the subsequent study. The gRNA with highest editing was selected for the subsequent HR assays. For ssDNA target design, typically the mutation site was positioned at the center flanked by 67-nt to 30-nt on each side (Fig. 1C). To measure homologous recombination efficiency, the ssDNA target was co-transfected with Cas9 RNPs into cells via electroporation. The genomic loci were PCR-amplified using the corresponding primers and then subjected to GeneArt® Genomic Cleavage Detection assay (ThermoFisher, USA) for restriction enzyme digestion.

### Culture of single cell derived clones

Transfected cells were washed with 500 μL of PBS buffer (Corning, USA) and resuspended at the density of 8 cells/mL with a total volume of 50 mL. 100 μL of the cell suspension was transferred into the wells of the 96 well tissue culture plates to ensure each well contained a single cell. The plates were incubated at 37 °C, 5% CO_2_ incubator (Thermo Scientific, USA). The plates were then scanned for single cell colonies as soon as small aggregates of cells are visible under a 4× microscope (usually after first week, depending on the growth rate of the cell line) to ensure the cell colonies were derived from a single cell. The cells were incubated for an additional 2–3 weeks to expand the clonal populations for further analysis and characterization.

### Harvest single cell derived clones

Single cell derived clones were washed with 100 µL of 1× PBS buffer (Corning, USA). 1 × 10^5^ of the cells from each clones were transferred into the PCR plate containing 25 µL “Direct lysis buffer”. The “Direct lysis buffer” was made by adding 10 μL of Proteinase K (Thermo Scientific, USA) to 1 mL DirectPCR Lysis Reagent (Thermo Scientific, USA). The PCR plate was incubated at 55 °C for 30 mins to lyse the cells and followed by 95 °C for 45 mins to denature the Proteinase K.

### HLA gene amplification and next generation sequencing

Multiplex long-range PCR were employed using AllType NGS assay (One Lambda, USA) to co-amplify 11 HLA loci including HLA-A, -B, -C, -DRB1,3,4,5 -DQB1 -DPB1, -DQA1 and -DPA1. HLA-A, -B, -C, -DQA1, and -DPA1 were amplified from 5′UTR to 3′UTR, and remaining loci are beginning at intron 1 to 3′UTR. Library construction was automated on the Biomek FX (Beckman coulter, USA). Sequence-ready libraries were validated and quantitated on the High Sensitivity D1000 ScreenTape (Agilent Technologies, USA) to allow for library normalization and equimolar pooling of all study samples on the Biomek FX (Beckman coulter, USA). Pooled libraries were diluted and loaded at Ion Chef (Thermo Scientific, USA) for template amplification. Sequencing on Ion S5 XL is followed the manufacture instruction (Thermo Scientific, USA). When the sequencing was done, the TSVEngine v1.2.0 (One Lambda, USA) was employed to analyze the data.

### Single antigen antibody testing

Neat and serum at 1:2 dilution were treated with DTT and tested for HLA antibodies using the IgG-SAB Assay from One Lambda (Canoga Park, CA) as previously described^44^. Antibodies were considered positive if the MFI >1000 for HLA-A, -B, -DR, -DQ and >2000 was used for HLA-C and -DP to correct for the enhanced amount of HLA-C and -DP antigens conjugated to the Luminex beads compared to the lower cell surface expression on lymphocytes.

### Flow cytometric analysis of HLA protein expression

Expression of HLA-B locus antigens in the edited cell lines containing the HLA-B*38:68Q allele and control cells were determined by flow cytometry using a FITC-conjugated monoclonal anti-IgG Bw4 antibody (One Lambda, USA). Approximately 10^5^ cells were incubated with 0.5, 1, and 2 μL (10 mg/mL) anti-Bw4 monoclonal antibody on ice for 30 mins. Isotype control cells were incubated with 0.5 μL of FITC-conjugated mouse IgG secondary antibody (Jackson ImmunoResearch, USA). After staining, cells were washed with 1× PBS buffer (Corning, USA) containing 2% FBS (Omega, USA) and suspended in 300 μL of PBS/2% FBS. TEM665 homozygous HLA-B*38:01:01:01 EBV transformed B cell line was used as the positive control, AOH749 and K562 were used as the negative control cells. Samples were tested in triplicate. Analysis of HLA B locus expression was performed using FlowJo software version 10 (BD, USA).

Expression of *HLA-B38* in the edited cell lines containing the HLA-B*38:68Q allele was determined using UCLA serum exchange sample that contains anti-B38 antibody but lacks Bw4 activity. Approximately 1.5 × 10^5^ cells/tube were incubated with 25 μL UCLA serum exchange serum at neat and 1:2 dilution for 30 mins at room temperature. After incubation, cells were washed 4 times with PBS/2% FBS followed by labeling for 20 mins at 4 °C with an anti-human IgG FITC-conjugated antibody (Jackson ImmunoResearch, USA). Negative sera routinely used in the clinical lab were used as controls. Samples were tested in triplicate. Analysis of *HLA-B38* expression was performed using FlowJo software version 10 (BD, USA).

### IFN-γ induced HLA-B*38:68Q cell surface expression

The HLA-B*38:68Q homozygous cell line and TEM665 HLA-B*38:01:01:01 homozygous EBV transformed B cell line (served as positive control) were pretreated with 500 U/mL of IFN-γ (R&D Systems, USA) for 48 h. The expression of HLA-B expression of the IFN-γ treated and untreated cell lines were determined using monoclonal anti-IgG Bw4 antibody (One Lambda, USA) by flow cytometry. A FITC-conjugated anti-mIgG (BioXCell, USA) was used as an isotype control. Samples were tested in triplicate. Analysis of expression was performed using FlowJo software version 10 (BD, USA).

### Statistical analysis

Each experiment of protein expression was tested in triplicate and the expression level is shown as mean fluorescence intensity (MFI) ± SD. Statistical analysis was performed the Student’s *t* test or ANOVA on GraphPad Prism 7 (GraphPad, USA). *P* < 0.05 was denoted as significant.

## Supplementary information

Supplementary Information

## Data Availability

All data generated or analyzed during this study are included in this article.
