# Supplementary material for: Integrate CRISPR/Cas9 for protein expression of HLA-B*38:68Q via precise gene editing
Source: Sci Rep. 2019 May 30;9:8067. doi: 10.1038/s41598-019-44336-7 (PMC6542842; doi:10.1038/s41598-019-44336-7)
Supplement: Supplementary file 1 — Supplementary Information [file 41598_2019_44336_MOESM1_ESM.pdf]

Integrate CRISPR/Cas9 for protein expression of *HLA-B*38:68Q* via precise gene editing

Yuxin Yin[1], Elaine F. Reed[1], and Qiuheng Zhang[1†]

[1]UCLA Immunogenetics Center, Department of Pathology & Laboratory Medicine, Los Angeles, 90095, USA

[†]Corresponding author: jqzhang@mednet.ucla.edu

Supplemental Table 1. Homozygous recombination efficiency testing[a] using different electroporation conditions in an *HLA-B*38:01:01:01* homozygous EBV transformed B cell line.

Program[b]	% indel[c]	Voltage	Width (ms)	Pulses
1	4.14%	0	1	1
2	35.47%	1400	20	1
3	45.95%	1500	20	1
4	49.85%	1600	20	1
5	58.41%	1700	20	1
6	19.19%	1100	30	1
7	28.29%	1200	30	1
8	42.27%	1300	30	1
9	4.75%	1400	30	1
10	15.40%	1000	40	1
11	27.28%	1100	40	1
12	36.66%	1200	40	1
13	20.29%	1100	20	2
14	32.98%	1200	20	2
15	45.74%	1300	20	2
16	47.66%	1400	20	2
17	9.89%	850	30	2
18	16.34%	950	30	2
19	27.83%	1050	30	2
20	40.51%	1150	30	2
21	30.96%	1300	10	3
22	40.18%	1400	10	3
23	49.39%	1500	10	3
24	58.39%	1600	10	3

[a]This testing is based on a control gRNA of *HPRT* (ThermoFisher)
[b]Program refer to Neon (ThermoFisher)
[c]Indel: insertion and deletion

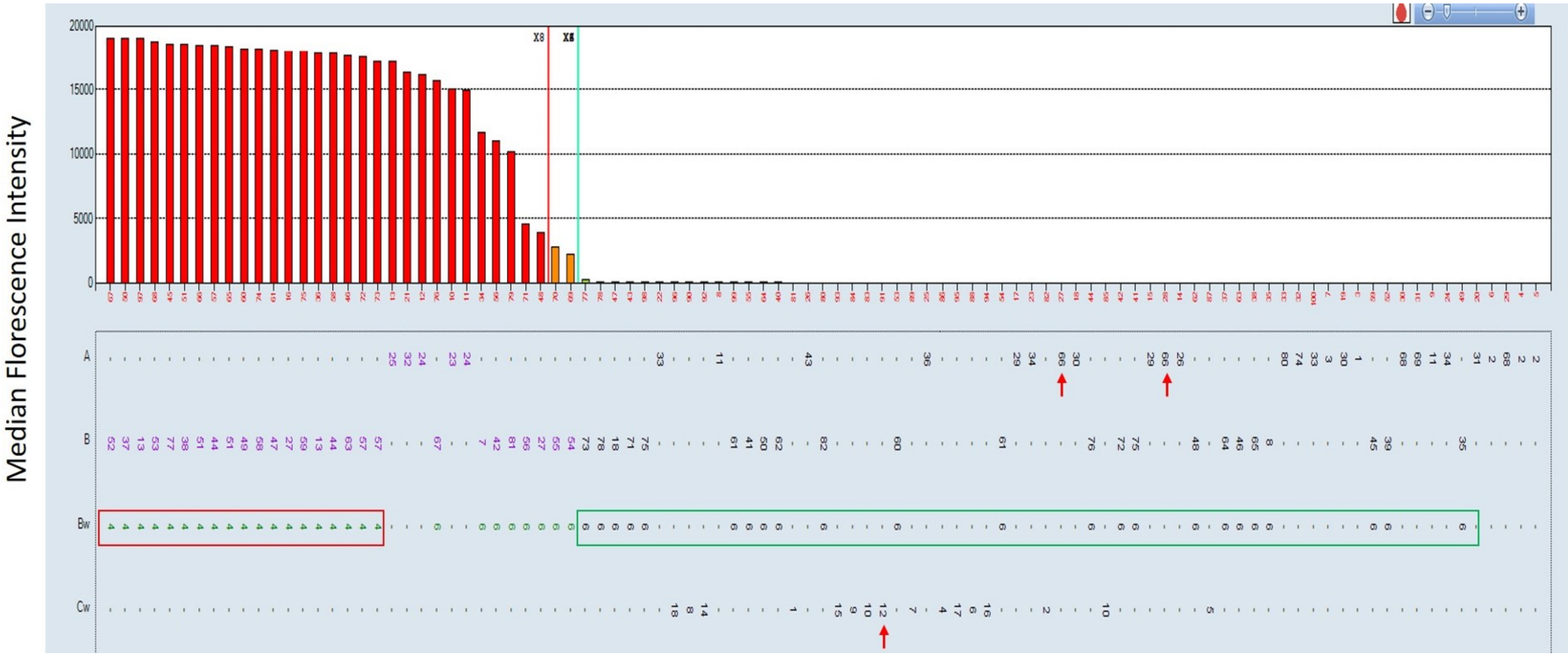

Supplemental Figure 1. Binding of monoclonal anti-Bw4 antibody to HLA by SAB class I test. The SAB class I test shows that the monoclonal anti-Bw4 antibody does not react to A66 (red arrow), C12 (red arrow), and Bw6 (green box) antigens, but reacts to Bw4 (red box) antigen carried by the *HLA-B*38:01:01:01* homozygous cells.

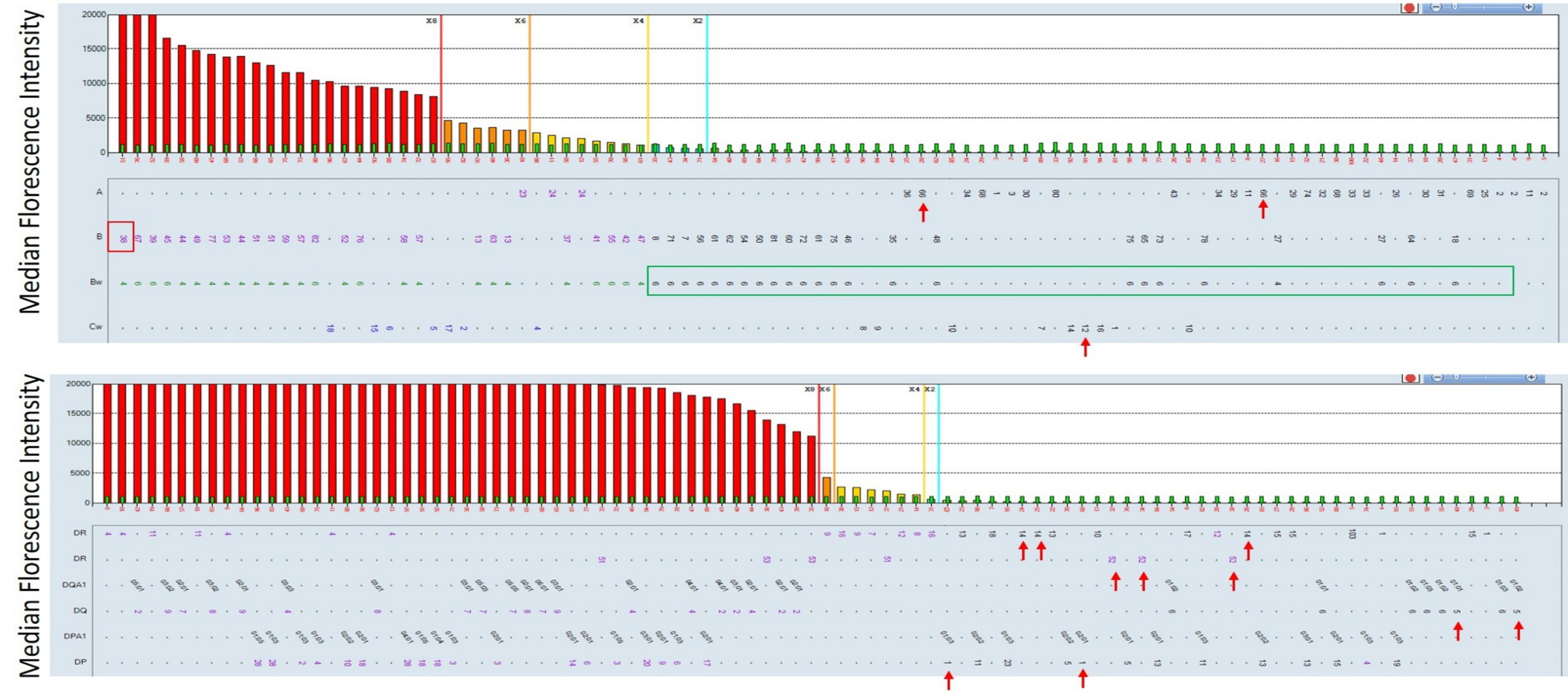

Supplemental Figure 2. Binding of polyclonal anti-B38 antibody to HLA antigens using SAB class I and class II test. The SAB class I and class II test shows that the polyclonal anti-B38 antibody does not react to A66 (red arrow), C12 (red arrow), Bw6 (green box), DR14 (red arrow), DR52 (red arrow), DQ5 (red arrow), and DP1 (red arrow) antigens, but reacts to B38 (red box) antigen carried by the *HLA-B*38:01:01:01* homozygous cells.
